# Impact of an education-centered medical home on quality at a student-volunteer free clinic

**DOI:** 10.1080/10872981.2018.1505401

**Published:** 2018-10-21

**Authors:** Abigail E. Russi, Smitha Bhaumik, Jackson J. Herzog, Marianne Tschoe, Andrea C. Baumgartner

**Affiliations:** a Northwestern University Feinberg School of Medicine, Chicago, IL, USA; b Department of Medicine, Northwestern University Feinberg School of Medicine, Chicago, IL, USA

**Keywords:** Longitudinal clerkships, primary care, student-run free clinic, undergraduate medical education, preventive medicine

## Abstract

**Background**: The continuity provided by longitudinal clerkships has documented benefits to medical student education. Yet, little quantitative data exist on the association between longitudinal clerkships and patient outcomes.

**Objective**: This study compares screening metrics of a longitudinal clerkship called the education-centered medical home (ECMH) with the standard clinical model at a student-volunteer free clinic (SVFC). In the ECMH model, the same attending physician staffs one half-day of clinic with same group of students weekly for 4 years. Standard clinical models are staffed with students and physicians who come to the SVFC based on availability.

**Design**: ECMH students aimed to increase human immunodeficiency virus (HIV) screening rates in their patient panel as part of a quality improvement project. Students prepared individualized care plans prior to patient visits that included whether screening had been performed. They were also reminded to confirm completion of testing. Percentages of patients screened for HIV before and after establishment of the ECMH were compared with four standard clinical models. Screening rates for breast, colon, and cervical cancer, as well as hepatitis C, served as secondary endpoints.

**Results**: While screening rates were initially similar between models (43.2% and 34.8% for the ECMH and standard clinical panels, respectively, *p* = 0.32), HIV screening rates increased from 43.2% to 95.0% in the ECMH compared with a significantly smaller increase from 35.0% to 50.0% in the standard clinical panel (*p* < 0.0001). Additionally, the ECMH resulted in statistically significantly increased screening rates for cervical cancer (*p* < 0.001) and hepatitis C (*p* < 0.0001).

**Conclusions**: This study demonstrates an association between a longitudinal ECMH clerkship and improved quality metrics at an SVFC. Even measures not targeted for intervention, such as colorectal cancer and hepatitis C, showed significant improvement in screening rates when compared with the standard clinical model.

## Introduction

Evidence suggests that longitudinal clerkships are well received by undergraduate medical students and improve patient centeredness and relationships. Students in continuity clerkships consistently report increased confidence with providing care to high-risk patients [1,2], increased responsibility and ownership for patient care [1–5], and increased satisfaction with the continuity experience for learning and receiving feedback when compared with block clerkships [6,7]. Additionally, students in continuity clerkships performed better than their peers on preceptor assessments and in-training evaluations while performing similarly on standardized exams [6,8].

While the benefits to student learning in longitudinal clerkships have been documented, patients could also gain from the continuity inherent in this model. Qualitative data from longitudinal integrated clerkships demonstrate that students who have ongoing relationships with patients make meaningful contributions to their care [5]. In addition, longitudinal clerkships empower students to learn about systems improvement and act upon what they have learned [9]. This sets the expectation of quality assessment and continued improvement at an early stage in trainees’ education. In these ways, longitudinal clerkships can improve the quality of medical care while providing students with a valuable experiential learning experience, a concept known as value-added medical education [10].

Despite multiple studies that have shown the educational benefits of longitudinal clerkships, little quantitative data exist about their impact on patient care. In 2015, Henschen et al. demonstrated improvements in cancer screening rates after implementation of a longitudinal clerkship called the education-centered medical home (ECMH) [3]. An ECMH is based on principles of the patient-centered medical home, including ‘continuity with a personal physician; team-based care; care coordination and integration; quality and safety; and enhanced access to care’ [11]. ECMH students attend clinic twice a month with the same preceptor for 4 years and engage in annual quality improvement (QI) projects. These activities allow them to become well acquainted with their patients and with the clinic’s operations.

In 2014, Northwestern University Feinberg School of Medicine (FSM) partnered with CommunityHealth Chicago (CHC) to pilot one of its student-volunteer free clinics (SVFCs) as an ECMH. In this study, we investigated the impact of the ECMH model on patient quality metrics compared with four standard clinical SVFC models at CHC. The primary difference between the two models is the clinic structure. SVFCs staff their clinics based on volunteer availability, whereas the ECMH schedules its clinic with the same supervising physician and a constant group of students. We hypothesized that the continuity of the ECMH model would allow for improved human immunodeficiency virus (HIV) screening compared with the standard clinical SVFC models at CHC.

While successful quality initiatives have been reported by other SVFCs [12–14], our study is the first to directly compare the ECMH model to multiple standard clinical SVFC models at the same free clinic site. HIV screening rates served as the primary outcome measure given that CHC had set a clinic-wide goal of increasing the rate of HIV screening for all patients across all clinics. Additionally, HIV screening is guideline based, often relies on patient education, and is influenced by the patient–physician relationship. We also investigated as secondary outcomes the ECMH’s impact on additional preventive care measures that were not actively tracked during the study period, specifically the screening rates for cervical cancer, colon cancer, breast cancer, and hepatitis C.

## Materials and methods

### Study Setting

CHC is the largest volunteer-based free clinic in the nation. It houses clinics from several Chicago-area medical schools and residency programs. Each institution, under the direct supervision of attending physicians, is responsible for the staffing and operations of its own clinic. CHC offers comprehensive medical care at no cost to uninsured individuals who do not qualify for federal or state programs. Funding support comes from individual, foundational, corporate, and institutional donors but not from government programs or grants.

### Two Types of Clinic Models

Two types of student clinics exist at CHC – the ECMH model and the standard clinical SVFC model. Patients were randomly assigned to each of the five clinics in this study.

- *The ECMH Model* – The ECMH consists of a core team of 16 medical students (4 from each year of study) and 1 attending physician. When randomly empaneled into the ECMH, patients are exclusively scheduled with their ECMH team and no other standard clinics at CHC. Clinic is held once weekly, and students attend twice per month on average. Once assigned to an ECMH, students are expected to remain at that site throughout medical school. Under the supervision of the attending physician, student teams are responsible for developing an individualized plan for assigned patients prior to clinic (prework), evaluating the patient, updating the electronic medical record (EMR) on the day of clinic, and following up on lab and diagnostic results after the visit. When possible, student teams then follow this assigned patient for all subsequent visits. Pre- and post-clinic huddles are held with discussions of case presentations, student-led presentations of relevant topics, and laboratory/imaging review sessions. In addition, each ECMH group is required to implement one QI project annually.
- *Standard Clinical SVFC Model* – Teams consist of medical students and licensed attending physician(s) responsible for patient care. Staffing occurs on an ad hoc basis, with students and supervising physicians coming to the SVFC based on their availability. Students from each class are allowed to come to their medical school clinic, but a minimum attendance is not required. Patients are rarely preassigned prior to clinic, so students are not required to perform prework. There are no pre- or post-clinic huddles. Standard clinical SVFCs are educated and participate in QI initiatives implemented by CHC but do not have their own specific targets.

### Patient Population

To qualify for care at CHC, patients must have no health insurance nor qualify for private or government-run insurance programs. They also must establish that their income does not exceed 250% of the Federal Poverty Level. Income verification is conducted annually for all patients.

### Inclusion Criteria

Patients seen at least once by the ECMH or four standard clinical SVFCs from November 2014 to May 2016 were included in this analysis. Further inclusion criteria centered on the screening and preventative care measures, specifically:

- HIV screening: Patients, aged 18–65 years old, were considered eligible [15].
- Cervical cancer screening: Female patients, aged 30–64 years old, were considered eligible [16].
- Colorectal cancer screening: Patients, aged 50–74 years old, were considered eligible [17].
- Breast cancer screening: Female patients, aged 50–74 years old, were considered eligible [18].
- Hepatitis C screening: Patients born between 1945 and 1965 were considered eligible [19].

### Study outcomes defined

- HIV screening: Documented result of HIV testing [15].
- Cervical cancer screening: Documented Pap smear within the past 3 years or a documented Pap smear with co-testing for high-risk human papillomavirus within the past 5 years [16].
- Colorectal cancer screening: Documented fecal occult blood immunoassay of a stool sample in the last year or colonoscopy in the previous 10 years [17].
- Breast cancer screening: Documented mammogram in the last 2 years [18].
- Hepatitis C screening: Documented result of hepatitis C antibody testing [19].

### Study Procedure

The proportion of patients screened for HIV was measured using the ‘Quality Report’ function in the Athena® EMR. This report details the HIV screening compliance, but not actual HIV status, of every patient coded in selected panels on the reporting date. After the results are received from an ordered HIV screening test, the EMR automatically considers this patient ‘satisfied.’ This quality metric can also be manually satisfied if documentation of screening is obtained elsewhere. If there is no documented HIV status, then the EMR marks this patient as ‘unsatisfied.’

Starting in November 2014, HIV screening reports were generated for patients empaneled in FSM’s ECMH clinic for 5 consecutive months and intermittently thereafter until May 2016. Quality reports from the four other Chicago-area medical school SVFCs were generated at five random intervals during the observation period from November 2014 to May 2016.

As part of the ECMH model’s QI project, the individualized plan for each patient included information about HIV screening status prior to the visit. Students were also reminded during team huddles to confirm completion of testing. Information about HIV screening status was obtained from the ‘Quality’ section of the Athena® EMR. This section tracks various guideline-specific preventive health measures for every patient. All clinics used the Athena® EMR during the study period. CHC provided guidance and resources for obtaining verbal consent for HIV testing to all clinics. Multilingual patient education materials were also provided by CHC. Rates of HIV screening achieved by each clinic were internally benchmarked on a monthly basis.

### Data Analysis

All data analyses were performed using GraphPad Prism 6.0. For comparison of categorical variables (sex, satisfied/not satisfied of quality metrics), a Fisher’s exact test was performed. For continuous measures (age), a Student’s *t* test was performed. To compare HIV screening rates between panels, a nonlinear line of best fit was calculated for each data set using a semilog function. Lines were compared by Comparison of Fits. For all statistical analyses, a two-tailed *p* value of less than 0.05 was considered significant.

## Results

At the conclusion of the 18-month study period, the patient age in the ECMH panel (*M* = 46.40, SD = 11.18) and standard clinical model (*M* = 45.95, SD = 11.34) did not differ. The sex demographics also did not differ between those empaneled in the ECMH compared with those followed by the standard clinical SVFCs (Table 1).

**Table 1.** Patient demographics of the ECMH and standard clinical panels who qualify for HIV screening.

	ECMH^a^ *N* (%)	Standard clinical SVFC^b^ *N* (%)
Age^c^		
≤30	11 (14)	34 (11)
31–40	14 (18)	65 (21)
41–50	26 (33)	87 (28)
51–60	23 (29)	98 (31)
>60	6 (8)	31 (10)
Sex^d^		
Male	32 (40)	171 (46)
Female	48 (60)	186 (54)

^a^Total number of ECMH patients = 80. ^b^Total number of standard clinical SVFC patients = 346. ^c^*p* > 0.05, based on Student’s *t* test (*t*(393) = 0.32, *p* > 0.05). ^d^*p* > 0.05, based on *χ*^2^ test (*χ*^2^(1, *N* = 436) = 1.69, *p* > 0.05).

At the start of the study period (November 2014), 16 of the 37 (43.2%) patients empaneled in the ECMH had documented HIV screening in the EMR, mirroring the national average of 40.8% [20]. As illustrated in Table 2, the total number of ECMH patients increased from 37 to 80 over the 18-month observation period. At the end of the study, the number of patients with a documented HIV status increased to 76 of 80 patients (95.0%). This was a significant increase compared with the pre-intervention screening rates (*p* < 0.0001).

**Table 2.** Number of patients screened for HIV in the ECMH panel during the 18-month observation period.

	ECMH		Standard clinical SVFC	
Date Month and year	Satisfied^e^ *N* (%)	Unsatisfied^f^ *N* (%)	Satisfied^e^ *N* (%)	Unsatisfied^f^ *N* (%)
November 2014	16 (43)	21 (57)	86 (35)	161 (65)
December 2014	23 (59)	16 (41)		
January 2015	27 (71)	11 (29)		
February 2015	30 (71)	12 (29)		
March 2015	39 (75)	13 (25)	119 (38)	194 (62)
May 2015	48 (84)	9 (16)		
July 2015	58 (81)	13 (19)		
August 2015	62 (84)	12 (16)		
September 2015	65 (83)	13 (17)		
March 2016	72 (92)	6 (8)	148 (47)	167 (53)
May 2016	76 (95)^g^	4 (5)	173 (50)	173 (50)

^e^Patients with a documented HIV screening test in the EMR. ^f^Patients without a documented HIV screening test in the EMR. ^g^*p* < 0.0001 by *χ*^2^ test (*χ*^2^(1, *N* = 117) = 40.34, *p* < 0.0001) compared with November 2014.

**Figure 1.**
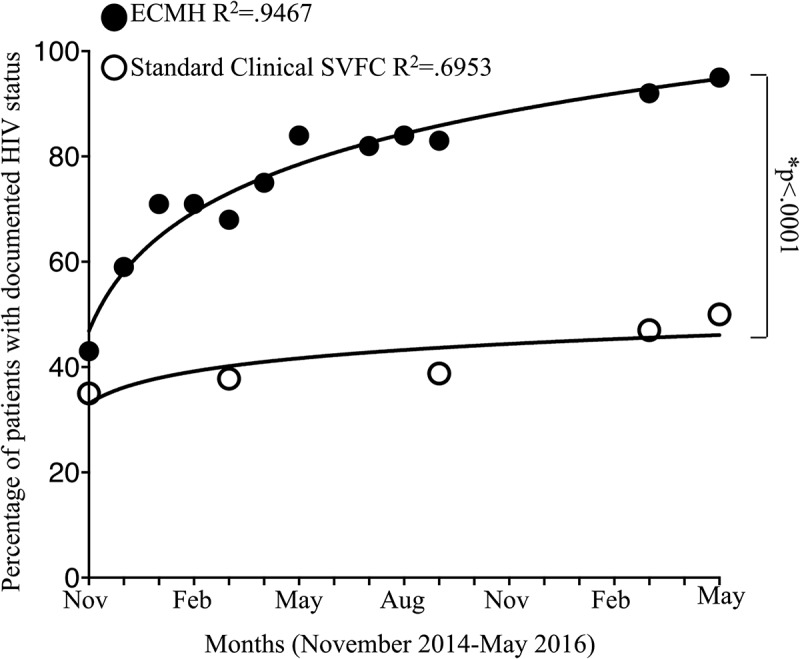
Comparison of HIV screening rates between the ECMH and standard clinical SVFC models during the 18-month observation period. The semilog line of best fit for screening rates per month was determined for the ECMH-modeled clinic and the standard clinical SVFCs. *p < 0.0001 by comparison of fits (*F*(2,13) = 187.5, *p* < 0.0001).

HIV screening rates for the ECMH and standard clinical patient panels are illustrated in Figure 1. Eighty-six of 247 (34.8%) in the standard clinical model had a documented HIV test at the start of the study period. At the end, 173 of the 346 (50.0%) had been screened for HIV. While screening rates were initially similar between models (43.2% and 34.8% for ECMH and standard clinical panels, respectively [*p* = 0.32]), screening rates increased from 43.2% to 95.0% in the ECMH panel compared with a significantly smaller increase from 35.0% to 50.0% in the standard clinical panel after the 18-month observation period (*p* < 0.0001).

Screening rates for secondary endpoints between the two models were compared at the conclusion of the study period (Table 3). Forty-seven of 52 (90.4%) patients were screened for cervical cancer in the ECMH panel versus 109 of 160 (68.1%) in the standard clinical model. Thirty-seven of 46 (80.4%) in the ECMH model were tested for hepatitis C, as opposed to 73 of 161 (45.3%) in the standard clinical model. Screening rates for breast and colon cancer were 65.6% and 68.1%, respectively, in the ECMH model. In the standard clinical model, breast and colon cancer screening rates were 47.9% and 54.9%, respectively. Overall, the ECMH model resulted in statistically significantly increased screening rates for cervical cancer (*p* < 0.001) and hepatitis C (*p* *<* 0.0001) and a trend toward increased screening for colorectal (*p* = 0.11) and breast cancer (*p* = 0.08) compared with the standard clinical model.

**Table 3.** Number of patients screened for secondary endpoints in the ECMH and standard clinical models during the 18-month observation period.

	ECMH		Standard clinical SVFC	
Screening test	Satisfied*N* (%)	Satisfied*N* (%)	*χ*^2^ test(df, *N*) = value	*p* Value
Cervical cancer	47/52 (90)	109/160 (68)	*χ*^2^(1, *N* = 212) = 10.00	<0.001
Colon cancer	32/47 (68)	95/173 (55)	*χ*^2^(1, *N* = 218) = 2.38	0.11
Breast cancer	21/32 (65)	45/94 (48)	*χ*^2^(1, *N* = 220) = 2.63	0.08
Hepatitis C	37/46 (80)	73/161 (45)	*χ*^2^(1, *N* = 207) = 17.69	<0.0001

## Discussion

This study examines the impact of an ECMH-modeled longitudinal clerkship on quality metrics when compared with standard clinical-modeled SVFCs. There was a statistically significant increase in the rate of HIV testing in the ECMH, both when compared with itself pre-intervention and with standard clinical SVFCs. Even quality metrics that were not the focus of the intervention, such as cervical cancer and hepatitis C, showed significant improvement compared with standard clinical SVFCs. This suggests that the ECMH model is a meaningful method for increasing preventive health measures overall and not just a chosen target such as HIV.

We speculate that improved HIV screening rates in the ECMH model resulted from its continuity among students, faculty, and patients. Supervision by the same preceptor provided students with consistent expectations for patient care. That consistency allowed for standardized processes, such as prework and huddles. Expectations were also easier to enforce through repetition and continued feedback. These characteristics are more difficult to maintain in a standard clinical SVFC where different students and faculty attend clinic each week.

In addition, continuity among ECMH students and their faculty preceptor potentially strengthened the team dynamic. Studies have shown that strong relationships in education can influence identity formation and motivation to learn [21]. In the ECMH model, interactions among students and faculty took place over a period of years rather than the weeks typical of most clerkships. Given this duration, participants had the time to establish comprehensive interpersonal relationships. Regular contact with learners at different levels allowed students to develop their skills as role models. Due to these factors, students may have felt more invested in the ECMH experience and fulfillment of objectives like the QI project.

Continuity between the ECMH and its patients likely engendered a commitment to care that contributed to improved outcomes. Longitudinal care is associated with an increased sense of responsibility among general practitioners toward their patients [22]. Furthermore, ECMH students have been shown to feel ownership for their patients [1]. Perhaps this model personalized a systems-based objective for the students, such that the QI project became not just a clerkship requirement but a means to improve the health of individual patients they knew through regular visits.

The improvement in secondary endpoints may be explained by both the ECMH model’s emphasis on system-based practice and the EMR’s organizational structure. The Athena® ‘Quality’ section facilitated tracking of preventive measures by housing them in one location for each patient chart. When students reviewed a chart to check HIV screening status, other unmet measures were highlighted by Athena®. Yet, the ‘Quality’ section alone cannot fully account for the improved outcomes. All clinics in this study had access to this feature, but the ECMH’s screening rates were higher than that of the standard clinical SVFCs. By requiring each site to complete an annual QI project, ECMH taught students about health-care quality by incorporating systems-based practice into its model. It also created common purpose by providing participants with a specific and actionable goal. Students who have previously tracked performance outcomes in other ECMH clinics have found that the QI process forced them ‘to consider clinical care at a new level they had not fully appreciated before’ [11]. Therefore, an increased appreciation for the importance of QI in our clinic may have led to improved outcomes in multiple endpoints rather than a single measure.

Important limitations must be considered when interpreting this data. Our results reflect patient outcomes over a short time period at one study site. The initially similar HIV screening rates between clinic models suggests that there were no biases in the starting patient populations. However, these findings may not be generalizable to other health-care settings and SVFCs. In addition, ECMH HIV screening rates were obtained more often than standard clinical SVFCs. Frequent monitoring was included in the ECMH’s QI project protocol to evaluate its progress and encourage accountability. We do not believe this affected the final outcomes given that screening of patients is recommended only once in an average risk population to identify those who are already HIV positive [15]. Although the age and sex of patients were similar between the two models, differences in other demographic variables – such as ethnicity, primary language spoken, or education level – were not examined. Lastly, differences in learners’ level of training could have affected outcomes. The ECMH model incorporated into its model an even distribution of students from each year of study. Standard clinical SVFCs were unable to do so because of the ad hoc nature of their scheduling process.

Our findings underscore the impact of value-added medical education in the ECMH-modeled longitudinal clerkship. The underserved populations seen at SVFCs and the students who care for them could truly benefit from the continuity of patient care, supervision, and systems that an ECMH provides. As the number of student-run clinics has increased over the past decades, they have been seen as an important supplement to an overburdened system of safety net health care [23]. However, the volunteer-based model on which these clinics are structured can affect the level of oversight [23,24] and the quality of preventive care delivered [25]. Our study demonstrates that an ECMH-modeled longitudinal clerkship could be a way to enhance the quality of health care for vulnerable populations and learners’ education concurrently. Given the impact of this study's findings, other medical schools may benefit from the implementation an ECMH-modeled longitudinal clerkship into their SVFCs. ECMH’s use of systems-based practice can help SVFCs accomplish their dual missions of education and service [26] while introducing students to core competencies [27] they will encounter during residency. This study supports the need for further research into the feasibility and impact of the ECMH model at other SVFCs. In addition, while potential benefit to straightforward screening metrics has been demonstrated, it should be investigated whether the ECMH-modeled longitudinal clerkship also affects outcomes associated with lifestyle modifications, such as smoking cessation, weight loss, or hemoglobin A1C values.

## Conclusion

These findings corroborate and extend upon a previous study [3] that showed that an ECMH-modeled longitudinal clerkship is associated with quantitative benefits to patient care. Our study further advances that work by demonstrating significantly increased screening rates for several measures in the ECMH model compared with standard clinical SVFCs, including those that were not specifically targeted for intervention. Future studies are needed to determine the full extent to which the ECMH model clinic benefits both student education and patient outcomes.
